# Long-Term Poverty, Rurality, and Geographic Disparities in Colorectal Cancer: A Spatial Analysis in Texas

**DOI:** 10.21203/rs.3.rs-9359859/v1

**Published:** 2026-04-27

**Authors:** Ryan Ramphul, Tracey Farrigan, Yixiao Chen, Yanchen Liu, Jooyeon Lee, Ruth Rechis, Karen Basen-Engquist, Lorna McNeill

**Affiliations:** The University of Texas Health Science Center at Houston; United States Department of Agriculture; The University of Texas Health Science Center at Houston; The University of Texas Health Science Center at Houston; The University of Texas Health Science Center at Houston; Cancer Prevention and Research Institute of Texas; The University of Texas MD Anderson Cancer Center; The University of Texas MD Anderson Cancer Center

## Abstract

**Purpose:**

Colorectal cancer (CRC) remains a leading cause of cancer morbidity and mortality in the United States and showed substantial geographic variation. Socioeconomically disadvantaged communities experience elevated CRC incidence, yet few studies have analyzed multi-decade poverty measures for long-term structural disadvantage.

**Methods:**

We analyzed CRC incidence from 2017–2021 across Texas using Texas Cancer Registry data. Age-sex-race/ethnicity standardized cases were incorporated as offsets in Bayesian spatial models to generate tract-level posterior mean relative risks. We evaluated four tract-level poverty measures (high poverty in 2019; persistent poverty since 1990; enduring poverty since 1980 and 1970), Rural-Urban Commuting Area (RUCA) classifications, and CRC screening rates among adults aged 50–75 years.

**Results:**

Long-term poverty and rurality were independently associated with elevated CRC risk. Compared with urban tracts, micropolitan and rural tracts had 7–8% higher risks. Poverty measures defined over longer durations showed stronger associations with CRC incidence: high poverty in 2017–2021 (RR = 1.04, 95% CrI: 1.01–1.07), persistent poverty since 1990 (RR = 1.04, 1.00–1.08), enduring poverty since 1980 (RR = 1.06, 1.01–1.11), and enduring poverty since 1970 (RR = 1.09, 1.04–1.15). Higher tract-level CRC screening prevalence was modestly associated with lower CRC incidence (RR per 1% increase = 0.99).

**Conclusion:**

Long-term poverty and rural residence were associated with elevated CRC risk in Texas, even after adjusting for screening prevalence. Multi-decade poverty measures and updated RUCA classifications identified geographic disparities not captured by single-year poverty indicators and supported geographically targeted screening, prevention, and resource allocation efforts.

## 1. Introduction

Colorectal cancer (CRC) remains a significant public health concern in the United States. It is the third most commonly diagnosed cancer among both men and women and the second leading cause of cancer-related death in both sexes combined [1, 2]. In 2025, an estimated 154,000 Americans will be diagnosed with CRC and approximately 52,900 will die from the disease [1, 2]. Over recent decades, overall CRC incidence and mortality rates have declined, largely due to improved screening and treatment. Between 2012 and 2021, incidence decreased by about 1% per year, and mortality declined steadily among older adults [1, 2]. However, these overall gains have not been uniform. Rates of CRC among adults under 50 years have increased by approximately 2.4% annually in recent years, underscoring the persistence of significant disparities in CRC burden across socioeconomic and geographic populations [1, 2].

Poverty is a well-recognized structural determinant of health, and long-term poverty represents a chronic, intergenerational form of socioeconomic deprivation that shapes community health across generations [3, 4]. The U.S. Department of Agriculture (USDA) defines persistent poverty as areas where at least 20% of residents have lived below the federal poverty line for three or more consecutive decades [5]. Approximately 10–12% of U.S. counties meet this criterion [6]. Communities facing long-term poverty experience cumulative disadvantages in infrastructure, education, healthcare access, and economic opportunity, which reinforce cycles of disinvestment and poor health [7, 8]. Recognizing these enduring inequities, federal initiatives such as the 10-20-30 provision, introduced in the 2009 American Recovery and Reinvestment Act, specifically direct resources to counties with persistently high poverty [5]. These efforts reflect growing policy awareness that persistent poverty is not merely an economic condition, but a durable determinant of population health.

Place-based social and structural factors, including poverty, food insecurity, healthcare access, and rurality, play a substantial role in shaping CRC incidence, mortality, and stage at diagnosis. Rural populations, especially those classified using the USDA’s Rural-Urban Commuting Area (RUCA) codes, experience higher CRC incidence and mortality than their urban counterparts [9, 10]. These disparities stem in part from socioeconomic deprivation, geographic isolation, limited access to screening and specialist care, and higher rates of being uninsured [11, 12]. High-poverty and food-insecure communities are also more likely to experience late-stage CRC diagnoses and poorer survival outcomes, reflecting cumulative barriers such as limited health literacy, lower preventive care use, and environmental and dietary risk exposures [8, 13]. Moreover, persistent poverty and rurality often overlap, creating compounded risks that reinforce geographic disparities in cancer outcomes [9, 12, 14].

While socioeconomic disadvantage is a well-documented contributor to CRC disparities, the duration of poverty exposure remains an underexplored determinant in cancer epidemiology. Most prior studies have relied on single-year or cross-sectional poverty indicators [15, 16], overlooking the cumulative impact of multidecade deprivation. Incorporating temporal dimensions of socioeconomic disadvantage, capturing not just whether a community is poor but how long it has been poor, may offer deeper insight into structural inequities that influence cancer risk [8, 12].

To address this gap, the present study applies Bayesian spatial models to examine associations between colorectal cancer incidence, long-term poverty, and rurality at the census tract level in Texas. Using the USDA’s Poverty Area Measures, which trace community poverty levels back to 1970, and 2020 RUCA-based classifications, we assess how the duration of community poverty exposure and degree of rurality jointly pattern CRC risk. We hypothesize a dose-duration gradient, whereby longer exposure poverty is associated with higher CRC incidence, with risk increasing progressively from urban to micropolitan to rural tracts.

## 2. Methods

### 2.1. Data sources and study population

This study analyzed CRC incidence across all census tracts in Texas. Data on newly diagnosed CRC cases were obtained from the Texas Cancer Registry (TCR), a high-quality, population-based registry that combines active and passive surveillance and recently began participating in the National Cancer Institute’s Surveillance, Epidemiology, and End Results (SEER) [17] Program. We included individuals diagnosed between 2017 and 2021 with International Classification of Diseases, Tenth Revision (ICD-10) codes C18-C20, representing malignant neoplasms of the colon, rectosigmoid junction, and rectum. Only the first primary CRC diagnosis per individual was included. Individual-level TCR records containing diagnosis details, demographic characteristics, and residential addresses were securely geocoded behind the UTHealth Firewall and aggregated to 2020 U.S. Census tract boundaries. This study was reviewed and approved by both the UTHealth Houston Committee for the Protection of Human Subjects (IRB #: HSC-SPH-24–0164) and the Texas Department of State Health Services (DSHS) Institutional Review Board (IRB#: IRB_2024–025_20250324), which oversees use of Texas Cancer Registry data. All analyses complied with the ethical standards of these institutions.

### 2.2. Long-term poverty

Tract-level poverty measures were derived from the USDA Poverty Area Measures dataset, 2025 edition, which harmonizes decennial Census and American Community Survey (ACS) data to identify areas of persistent socioeconomic deprivation. These updated measures employ 2020 Census tract boundaries, improving spatial accuracy over prior publicly available versions that relied on 2010 census tract boundaries. We defined four tract-level poverty indicators:

- High poverty in 2021: Tracts with ≥ 20% of residents living below the federal poverty line in the 2021 ACS 5-year estimates.
- Persistent poverty since 1990: Poverty ≥ 20% in 1990, 2000, 2011, and 2021.
- Enduring poverty since 1980: Poverty ≥ 20% in 1980, 1990, 2000, 2011, and 2021.
- Enduring poverty since 1970: Poverty ≥ 20% in 1970, 1980, 1990, 2000, 2011, and 2021.

These multi-decade measures capture the duration of community deprivation, providing a more comprehensive assessment of socioeconomic context than single-year poverty estimates [18].

### 2.3. Urbanicity–rurality

Rural-urban status was assessed using the 2020 Rural-Urban Commuting Area (RUCA) codes [19], which classify tracts on a ten-point continuum from the most urban (1) to the most rural (10) based on population density, commuting flows, and economic integration [20, 21]. For interpretability and consistency with prior research, we collapsed these codes into three categories:

- Urban: RUCA 1–3
- Micropolitan: RUCA 4–6
- Rural: RUCA 7–10

The 2020 RUCA update, released in mid-2025, represents the first major revision of the classification since 2010. This update enables more accurate characterization of recent demographic and commuting shifts, making this study among the first to apply the updated RUCA system in a spatial epidemiologic analysis of cancer. Table 1 summarized the number of tracts classified as urban, micropolitan, and rural tracts in each poverty designation.

### 2.4. CRC screening rates among adults aged 50–75

The primary objective of this analysis was to estimate the association of rural–urban context (RUCA classification) and area-level persistent poverty with CRC risk. Although high body mass index (BMI), low physical activity, and cigarette smoking have been shown to be associated with increased CRC risk [22], these factors primarily reflect behavioral composition and their inclusion in disease-mapping models may mediate the associations between rurality, poverty, and CRC risk. Adjusting for such tract-level health behaviors would therefore attenuate the total effects that we are to analyze and thus excluded from our analyses.

In contrast, CRC screening prevalence was included as an adjustment variable, as screening has been shown to be effective in reducing CRC incidence through early detection and removal of precancerous lesions [23]. CRC screening also reflects access to preventive care that may vary across the tracts independent from rurality or poverty. Tract-level CRC screening prevalence estimates were obtained from the CDC PLACES dataset [24]. Although released in 2024, these modeled estimates are derived from pooled American Community Survey (ACS) 2018–2022 data, which closely aligns with the 2017–2021 CRC diagnosis period considered in this study. The preliminary analysis found no significant association between RUCA classification and screening prevalence; thus, both variables were retained as independent covariates in all models.

### 2.5 Outcome variable

The primary outcome was the number of newly diagnosed CRC cases per census tract during 2017–2021. Individual cases were stratified by sex, age, and race/ethnicity within census tracts and subsequently aggregated. Using a stratified population, age-sex-race/ethnicity-standardized expected counts were externally computed and incorporated as an offset in the model described below. Because our analyses were conducted at the tract level, the final case counts were aggregated to the tract level. The resulting model therefore estimates tract-level CRC risk relative to the underlying population at risk.

### 2.6. Statistical analysis

To model tract-level CRC incidence, we employed a Bayesian negative binomial spatial model with a Besag-York-Mollié 2 (BYM2) structure [25]. The negative binomial distribution was selected to accommodate overdispersion in count data. The BYM2 framework accounts for both spatially structured and unstructured random effects, allowing us to capture geographic clustering and unmeasured spatial heterogeneity across Texas tracts. The model is specified as:

$$\text{log}\left(\mu_{\text{i}}\right)=\text{log}\left({\text{E}}_{\text{i}}\right)+\backslash \text{varvec}{\text{x}}_{\text{i}}^{\text{′}}\backslash \text{varvec}\beta +\mu_{\text{i}}$$

where *μ*_*i*_ is the model-based mean of CRC cases in tract *i* under the full negative binomial model; *E*_*i*_ is the age-sex-race/ethnicity standardized offsets that are externally computed based on population counts in each stratum; \varvec*x*_*i*_ is a vector of tract-level covariates (e.g., poverty measures, RUCA category, CRC screening rate among adults age 50–75), \varvec*β* is a vector of regression coefficients, and *u*_*i*_ represents the combined spatial random effect (structured and unstructured components), where structured dependency was modeled using a conditional autoregressive (CAR) structure based on first-order queen contiguity method. Penalized complexity (PC) priors were used to induce shrinkage toward the base model, preventing excessive spatial variability.

We estimated four separate models, each including RUCA classification as a core covariate and differing by the poverty indicator, with adjustment for the tract-level CRC screening rate among older adults. The models are as follows:

- Model A: RUCA classification + high poverty in 2021 + CRC screening rates among adults aged 50–75
- Model B: RUCA classification + persistent poverty since 1990 + CRC screening rates among adults aged 50–75
- Model C: RUCA classification + enduring poverty since 1980 + CRC screening rates among adults aged 50–75
- Model D: RUCA classification + enduring poverty since 1970 + CRC screening rates among adults aged 50–75

In all models, “urban” served as the reference category for RUCA classification, and “not persistent” or “not enduring” tracts were the reference for poverty indicators. Relative risks (RRs) and 95% credible intervals (CrI) were computed for each covariate, with significance inferred when the CrI did not include RR of 1. Because U.S. Census tracts are designed to contain relatively stable populations, typically between 2,500 and 8,000 residents, with an ideal size of approximately 4,000, we restricted analyses to tracts with a minimum population of 1,000. Tracts below this threshold are atypical and in Texas primarily corresponded to non-residential or special-use areas (e.g., airports, industrial zones, ports, parks, or correctional facilities) with zero or near-zero resident populations. Inclusion of such tracts in small-area disease mapping can produce unstable or misleading risk estimates due to artificially inflated rates [26]. Only 88 of 6,896 census tracts (1.3%) were excluded from the analysis. Analyses were conducted in R version 4.2.2 using the Integrated Nested Laplace Approximation (INLA) approach for Bayesian inference [27].

## 3. Results

Descriptive characteristics of Texas census tracts are presented in Table 1. Across tracts, mean CRC incidence rates were higher in rural and micropolitan areas compared with urban areas. Poverty measures varied substantially across urbanicity categories, with persistent and enduring poverty more commonly observed in rural tracts. Screening prevalence among adults aged 50–75 years was generally lower in rural and high-poverty tracts, suggesting potential geographic clustering of structural disadvantage.

### 3.1. Urbanicity-rurality associations

Adjusted relative risk (RR) estimates from the four Bayesian negative binomial BYM2 models are shown in Table 2. Across all model specifications, urbanicity remained a consistent predictor of CRC incidence after adjustment for poverty measures and screening prevalence. Compared with urban tracts, micropolitan tracts exhibited 7–8% higher CRC risk (RR range: 1.074–1.079), while rural tracts showed similar excess risk (RR range: 1.069–1.075). All 95% credible intervals excluded 1.0, indicating statistically robust associations. These findings suggest that rural–urban disparities in CRC incidence persist independent of long-term poverty measures and screening differences.

### 3.2. Poverty measures

All poverty definitions were positively associated with CRC incidence after accounting for rurality and screening prevalence (Table 2). Effect estimates increased monotonically with longer poverty duration. Tracts classified as high poverty in 2021 had 4.2% higher CRC risk (RR = 1.042; 95% CrI: 1.012–1.073). Persistent poverty since 1990 was associated with 4.1% higher risk (RR = 1.041; 95% CrI: 1.004–1.08). Enduring poverty since 1980 corresponded to a 5.9% higher risk (RR = 1.059; 95% CrI: 1.012–1.109), while enduring poverty since 1970 demonstrated the strongest association (RR = 1.093; 95% CrI: 1.039–1.15). The increasing magnitude of effect estimates across poverty definitions suggests that longer-standing community-level deprivation is associated with progressively higher CRC incidence.

### 3.3. Screening rate adjustment

Higher tract-level CRC screening prevalence was modestly associated with lower CRC incidence across models (RR ≈ 0.992—0.993 per 1% increase; Table 2). Under the model’s log-linear specification, this corresponds to approximately an 8% lower risk per 10-percentage-point increase in screening prevalence. Although the per-unit effect was small, the cumulative impact at the population level may be meaningful.

### 3.4. Spatial Distribution of CRC Risk and Long-Term Poverty

**Figure 1:**
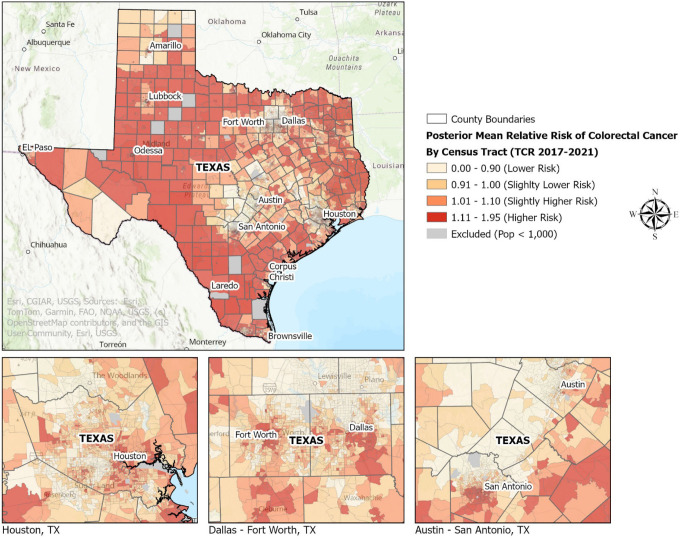
Posterior Mean Relative Risk of Colorectal Cancer from the Enduring Poverty Since 1970 Model (BYM2), Texas Census Tracts. Posterior mean relative risks (RR) of colorectal cancer estimated from the fully adjusted Bayesian negative binomial BYM2 model including RUCA classification, CRC screening prevalence, and enduring poverty since 1970. Values greater than 1.1 indicate higher CRC risk relative to the Texas statewide average. Estimates incorporate spatial smoothing to account for spatial autocorrelation and stabilize risk in sparsely populated tracts. Light gray areas represent census tracts excluded from analysis due to population <1,000. The map lines delineate study areas and do not necessarily depict accepted national boundaries.

**Figure 2:**
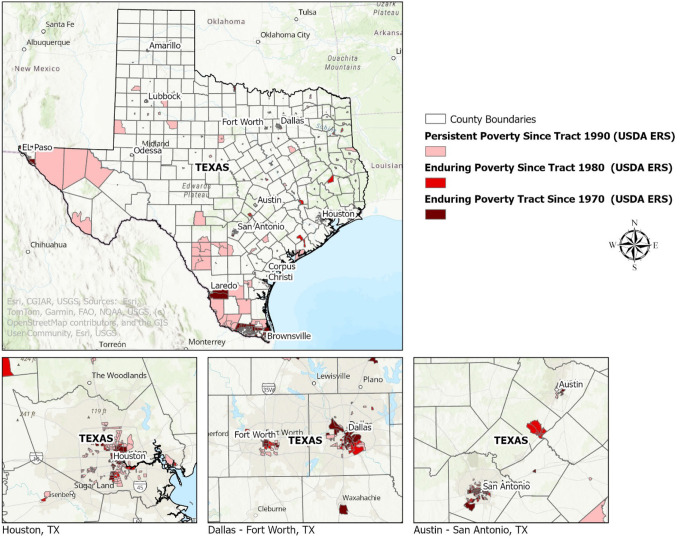
Geographic Distribution of Persistent Poverty Since 1990 and Enduring Poverty Since 1980 and 1970, Texas Census Tracts. Spatial distribution of census tracts classified as persistent poverty since 1990 and enduring poverty since 1980 and enduring poverty since 1970 based on USDA Poverty Area Measures (Farrigan & Sanders, 2024). The map lines delineate study areas and do not necessarily depict accepted national boundaries.

The spatial distribution of tract-level posterior mean relative risks from the fully adjusted model including enduring poverty since 1970 (Model D) is shown in Fig. 1. Elevated CRC risk was geographically clustered in several rural and persistently impoverished regions of Texas. The geographic distribution of census tracts classified as persistent poverty since 1990 and enduring poverty since 1980 and 1970 is presented in Fig. 2, illustrating the long-term structural deprivation patterns underlying the observed CRC risk gradients.

Posterior mean relative risks (RR) of colorectal cancer estimated from the fully adjusted Bayesian negative binomial BYM2 model including RUCA classification, CRC screening prevalence, and enduring poverty since 1970. Values greater than 1.1 indicate higher CRC risk relative to the Texas statewide average. Estimates incorporate spatial smoothing to account for spatial autocorrelation and stabilize risk in sparsely populated tracts. Light gray areas represent census tracts excluded from analysis due to population < 1,000. The map lines delineate study areas and do not necessarily depict accepted national boundaries.

Spatial distribution of census tracts classified as persistent poverty since 1990 and enduring poverty since 1980 and enduring poverty since 1970 based on USDA Poverty Area Measures (Farrigan & Sanders, 2024). The map lines delineate study areas and do not necessarily depict accepted national boundaries.

## 4. Discussion

In this statewide spatial analysis, both rural residence and long-term tract-level poverty were associated with higher CRC incidence. The model incorporating enduring poverty since 1970 demonstrated the strongest poverty-CRC association, independent of rurality and screening prevalence. These findings indicate that longer-duration area-level poverty corresponds to greater CRC burden across Texas census tracts.

These results align with prior research documenting socioeconomic and geographic disparities in CRC. Lower neighborhood socioeconomic status has been associated with higher CRC incidence and mortality [28], and rural residence has been linked to reduced screening access and poorer outcomes [9, 10]. County-level analyses further show that overall cancer mortality is higher in persistently poor counties [8, 12]. By leveraging census tract-level data and multi-decade poverty measures, our study extends this literature and provides greater spatial precision than prior county-level or cross-sectional analyses [15, 16, 29]. The spatial patterns observed in Fig. 1 reveal elevated CRC risk in rural and long-term impoverished regions of Texas, while Fig. 2 illustrates the geographic concentration of persistent and enduring poverty across the state. Together, these patterns demonstrate how multidecade area-level deprivation corresponds with contemporary CRC disparities.

Several mechanisms may explain the observed associations. Communities experiencing persistent poverty face chronic underinvestment in healthcare infrastructure, education, and economic opportunity [8, 12]. Such environments are associated with higher prevalence of CRC risk factors including poor diet, physical inactivity, tobacco use, and chronic stress [8, 22]. In rural areas, these structural challenges are compounded by geographic isolation, provider shortages, longer travel distances to screening facilities, and higher uninsured rates [10, 16]. Over decades, these intersecting structural disadvantages may produce cumulative contextual exposures that increase CRC incidence at the population level.

Importantly, the inclusion of tract-level screening prevalence suggests that geographic disparities in CRC incidence persist even after accounting for preventive care uptake. Although higher screening prevalence was associated with modestly lower CRC risk, screening differences did not fully explain the observed rural and long-term poverty gradients. This finding aligns with prior evidence that structural determinants influence cancer outcomes beyond individual-level screening behaviors [8, 12].

These findings have meaningful public health implications. Incorporating USDA Poverty Area Measures and updated 2020 RUCA classifications into cancer control planning may help identify high-risk communities for targeted intervention. Tracts experiencing enduring poverty and rural isolation may benefit from enhanced screening outreach, mobile colonoscopy services, mailed fecal immunochemical testing (FIT) programs, patient navigation services, and telehealth expansion. Such place-based strategies align with broader calls to address structural inequities as drivers of cancer disparities [3, 4].

This study has several strengths. The use of census tract-level data provides finer spatial resolution than county-level analyses, revealing localized disparities that might otherwise be obscured. The integration of multi-decade poverty measures spanning 1970–2021 captures temporal depth rarely incorporated in cancer epidemiology. Application of the updated 2020 RUCA codes enhances contemporary rural classification. Finally, the Bayesian BYM2 modeling framework accounts for spatial autocorrelation and overdispersion, yielding stabilized posterior mean risk estimates across the state [25, 26].

Nonetheless, limitations should be acknowledged. This ecological analysis cannot infer individual-level risk, and residual confounding may persist despite adjustment for screening prevalence. Screening rates derived from the CDC PLACES dataset are modeled estimates with associated uncertainty, which was not propagated into the spatial model. Additionally, migration and population mobility may influence long-term exposure classification, particularly in rapidly growing urban regions. Future research should explore multilevel designs linking individual-level screening, stage at diagnosis, and comorbidities with long-term neighborhood context. Expanding this framework to other states or incorporating environmental and food environment indicators may further elucidate pathways linking enduring poverty to CRC risk.

In conclusion, enduring poverty since 1970 and rural residence were independently associated with elevated CRC incidence across Texas census tracts. These findings reinforce the importance of incorporating temporal depth into socioeconomic measures and underscore the need for geographically targeted, equity-focused interventions to reduce CRC burden in persistently disadvantaged communities.

## Figures and Tables

**Table 1:** CRC Incidence rate per 100,000 stratified by rurality-urbanicity for each poverty term.

High poverty in 2019	*Urban*	*Micropolitan*	*Rural*
	131 (183)	145 (62.6)	190 (84.4)
**Low poverty in 2019**	124 (151)	152 (64.0)	183 (91.4)
*Number of tracts*	4313 (82.7%)	450 (8.6%)	453 (8.7%)
**Persistent poverty since 1990**	130 (70.9)	147 (65.3)	172 (75.5)
**Not Persistent poverty since 1990**	119 (55.7)	151 (62.9)	185 (81.8)
*Number of tracts*	4022 (82.3%)	432 (8.8%)	433 (8.9%)
**Persistent poverty since 1980**	139 (75.2)	151 (87.1)	148 (19.0)
**Not Persistent poverty since 1980**	117 (54.4)	138 (56.3)	176 (59.2)
*Number of tracts*	3906 (95.4%)	138 (3.4%)	51 (1.2%)
**Persistent poverty since 1970**	144 (76.0)	121 (57.6)	162 (0.0)
**Not Persistent poverty since 1970**	117 (55.0)	140 (59.8)	175 (58.8)
*Number of tracts*	3906 (95.4%)	138 (3.4%)	51 (1.2%)

Incidence rates are presented as mean (standard deviation). Number of tracts is presented as count (percentage).

**Table 2:** Relative risks (RRs) of colorectal cancer (CRC) incidence from Bayesian spatial models (BYM2), Texas census tracts, 2017–2021.

	Model A	Model B	Model C	Model D
	RR (95% CrI)	RR (95% CrI)	RR (95% CrI)	RR (95% CrI)
**Rurality-Urbanicity**				
Urban (ref)	1	1	1	1
Micropolitan	1.074 (1.022 1.128)*	1.074 (1.022 1.128)*	1.078 (1.025 1.132)*	1.079 (1.027 1.134)*
Rural	1.069 (1.021 1.119)*	1.07 (1.023 1.12)*	1.074 (1.026 1.124)*	1.075 (1.027 1.125)*
**Prevalence of CRC screening rates among adults aged 50–75 years**	0.993 (0.991 0.995)*	0.992 (0.991 0.994)*	0.992 (0.99 0.994)*	0.992 (0.991 0.994)*
**High poverty in 2017–2021 (Ref: Low poverty in 2017–2021)**	1.042 (1.012 1.073)*			
**Persistent poverty since 1990**		1.041 (1.004 1.08)*		
**(Ref: Not Persistent poverty since 1990)**				
**Enduring poverty since 1980**			1.059 (1.012 1.109)*	
**(Ref: Not Enduring poverty since 1980)**				
**Enduring poverty since 1970**				1.093 (1.039 1.15)*
**(Ref: Not Enduring poverty since 1970)**				

Models adjusted are adjusted for tract-level prevalence of obesity, cigarette smoking, and CRC screening rates among adults aged 50–75. Each model included a different poverty indicator: (A) high poverty 2017–2021, (B) persistent poverty since 1990, (C) enduring poverty since 1980, and (D) enduring poverty since 1970. For the robustness, we analyzed tracts that have at least 1,000 population.

* Indicates 95% Bayesian credible interval (CrI) for the relative risk excluding the null value of 1.

## Data Availability

The datasets generated and/or analyzed during the current study are not publicly available due to data use restrictions and the need to protect individual privacy. Texas cancer data have been provided by TCR, the Cancer Epidemiology and Surveillance Branch, Texas Department of State Health Services, 1100 West 49th Street, Austin, TX 78756. Data from TCR is supported by the following: Cooperative Agreement #1NU58DP007140 from the Centers for Disease Control and Prevention (CDC), Contract #75N91021D00011 from the National Cancer Institute’s Surveillance, Epidemiology, and End Results (SEER) Program, and the Cancer Prevention and Research Institute of Texas (CPRIT).
